# Determination of physiological parameters for endogenous glucose production in individuals using diurnal data

**DOI:** 10.1186/s42490-019-0030-z

**Published:** 2019-11-15

**Authors:** Mariël F. van Stee, Shaji Krishnan, Albert K. Groen, Albert A. de Graaf

**Affiliations:** 1 Netherlands Organisation for Applied Scientific Research (TNO), Utrechtseweg 48, Zeist, 3704 HE The Netherlands; 2 Amsterdam Diabetes Center and Department of Vascular Medicine Academic Medical Center, Meibergdreef 9, Amsterdam, 1105 AZ The Netherlands; 3 Department of Pediatrics, University of Groningen, University Medical Center Groningen, Hanzeplein 1, Groningen, 9713 GZ The Netherlands

**Keywords:** Endogenous glucose production (EGP), Non-linear system, Parameters, Diurnal information, Partial differentiation

## Abstract

**Background:**

Triple tracer meal experiments used to investigate organ glucose-insulin dynamics, such as endogenous glucose production (EGP) of the liver are labor intensive and expensive. A procedure was developed to obtain individual liver related parameters to describe EGP dynamics without the need for tracers.

**Results:**

The development used an existing formula describing the EGP dynamics comprising 4 parameters defined from glucose, insulin and C-peptide dynamics arising from triple meal studies. The method employs a set of partial differential equations in order to estimate the parameters for EGP dynamics. Tracer-derived and simulated data sets were used to develop and test the procedure. The predicted EGP dynamics showed an overall mean *R*^2^ of 0.91.

**Conclusions:**

In summary, a method was developed for predicting the hepatic EGP dynamics for healthy, pre-diabetic, and type 2 diabetic individuals without applying tracer experiments.

## Background

The plasma glucose concentration in healthy humans is strictly controlled. During fasting conditions the glucose is around 90 mg/dl and after food intake it can reach a concentration of 126-144 mg/dl [1]. The glucose comes into the bloodstream by means of absorption from the intestine, breakdown of glycogen (glycogenolysis) and gluconeogenesis [1].

The release of glucose resulting from glycogenolysis and gluconeogenesis is called the endogenous glucose production (EGP). The liver is the major contributor to EGP [2]. Within 1-2 h after a meal the EGP is suppressed to 30%-50% of its fasting value [3–6]. Glucose from the ingested meal will be taken up by the liver, brain, skeletal muscle and adipose tissue in the hours after a meal. The EGP will return to the fasting value after 4 h [7].

Understanding the mechanisms regulating glucose homeostasis is important to better comprehend the effects of new therapies for diseases such as diabetes [8]. Information about EGP dynamics is relevant, because the regulation of this important physiological process is changed in diabetic patients [9–11]. For instance, when fasting hyperglycemia develops in diabetic subjects, elevated rates are observed for both gluconeogenesis and glycogenolysis [12]. Increased gluconeogenesis activity might even be an early feature in the development of glucose dysregulation [13, 14].

Isotope tracer methods are used for the measurement of metabolic (e.g. glucose) flux rates [15], and have been used to investigate amongst others the glucose-insulin dynamics in organs/tissue (e.g. EGP) [16]. Glucose triple trace isotope labeling experiments can give reliable estimates of EGP, but are expensive and labour intensive [17, 18]. It is therefore not feasible to perform these experiments for large epidemiological and genetic studies. Therefore, we looked for alternative approaches to determine EGP dynamics that avoid the use of tracers.

Several mathematical models describing human glucose-insulin dynamics have been published in literature [19]. These models are constantly improved or extended to increase their predictive power. Man et al. (20) proposed a glucose dynamics model that describes the glucose and insulin fluxes during a mixed meal. This model is divided into various subsystems, such as gastrointestinal tract, liver, *β*-cell, muscle and adipose tissue. The various parameters of the model in [20] have been estimated from data from glucose isotope labeling experiments in normal humans and humans with type 2 diabetes (T2D). In [20], the EGP in the liver subsystem is described using 2 differential equations and 6 parameters and requires plasma glucose, plasma insulin and portal insulin dynamics data.

Although the model [20] includes parameters settings for normal and type 2 diabetic subject groups, it is of interest to be able to determine these parameters of individual subjects without the need for a new tracer experiment. In this paper, a mathematical procedure is described that is capable of calculating the glucose dynamics model parameters in the model [20] for the liver without glucose isotope labeling experimental data to estimate diurnal EGP dynamics.

## Results

The proposed mathematical procedure to estimate the parameters for EGP dynamics was tested on the 5 different data sets. The resulting predicted EGP time profiles were compared to the experimental ones. Residual plots for each data set display the deviation of the predicted EGP.

### EGP parameter estimates

Application of the proposed procedure to data from normal, pre-diabetic and diabetic subjects receiving diurnal mixed meals (data sets 1-5) resulted in parameter values reported in Table 1.

**Table 1:** EGP model parameter and mean coefficient of determination (*R*^2^) values for the healthy condition data sets (data set 1(*θ*_ds1_) and data set 2 (*θ*_ds2_)), pre-diabetic condition data sets (data set 3(*θ*_ds3_) and data set 4 (*θ*_ds4_)), and the diabetic condition data set (data set 5 (*θ*_ds5_)) determined using the proposed procedure

Name	*θ*_ds1_	*θ*_ds2_	*θ*_ds3_	*θ*_ds4_	*θ*_ds5_	Unit
k _*p*2_	0.0056 (0.0029)	0.0025 (0.0021)	0.0044 (0.0021)	0.0034 (0.0021)	0.0042 (0.0009)	min ^−1^
k _*p*3_	0.0109 (0.0119)	0.0109 (0.0093)	0.0049 (0.0047)	0.005 (0.0093)	0.0073 (0.0053)	mg/kg/min per pmol/l
k _*p*4_	0.075 (0.09)	0.045 (0.062)	0.03 (0.062)	0.051 (0.062)	0.037 (0.074)	mg/kg/min per pmol/kg
*A*	0.9 (0.8129)	0.90 (1.00)	0.85 (1.00)	0.88 (0.99)	0.88 (1.01)	dimensionless
*R*^2^**Breakfast**	0.95	0.90	0.97	0.93	0.93	
*R*^2^**Lunch**	0.86	0.91	0.97	0.84	0.90	
*R*^2^**Dinner**	0.92	0.91	0.97	0.85	0.92	

Values between brackets indicate the optimal parameter value obtained from directly fitting the experimental EGP data against the model (Eq. 20)

In particular, the estimated hepatic glucose effectiveness *k*_*p*2_ was found to have structurally higher values and the portal insulin signaling parameter *k*_*p*4_ structurally lower values compared to the true parameter values for all data sets. The hepatic glucose effectiveness parameter *k*_*p*2_ showed similar values when comparing simulated data sets (healthy, both pre-diabetic conditions (*θ*_ds3_ and *θ*_ds4_) and type 2 diabetic). The value of the estimated parameter *k*_*p*3_ in all data sets showed small deviations compared to the optimal parameter values. The parameter *A*, describing a changed insulin sensitivity during breakfast, showed to be smaller than 1 for all data sets. Parameter differences between the healthy data sets (data sets 1 and 2) and the diabetic data set (data set 5) were observed.

### Diurnal EGP dynamics

**Fig. 1:**
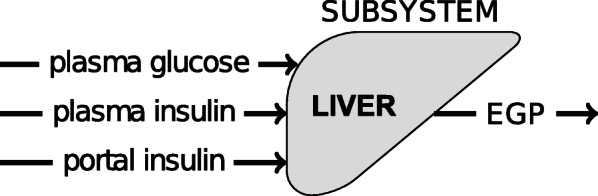
Schematic representation of the liver model in [20]. The inputs of the liver subsystem are the plasma glucose, plasma insulin and portal insulin concentrations. The output of the system is *EGP*

Figure 1 displays the predictions of diurnal EGP dynamics using the estimated parameter values (*θ*_ds1-5_) (Table 1) vs. the experimental data of data sets 1 to 5. The mean coefficient of determination (*R*^2^) for data set 1 was 0.91 and for data set 2 0.91. *R*^2^ values for pre-diabetic subjects were 0.97 (data set 3), and 0.87 (data set 4). The *R*^2^ for a type 2 diabetic subject was 0.92 (data set 5). The less well approximated EGP dynamics were seen in data set 1 and data set 4, consisting of healthy and pre-diabetic subjects. The EGP dynamics in data set 2 and 3 were most accurately predicted.

### Model performance

**Fig. 2:**
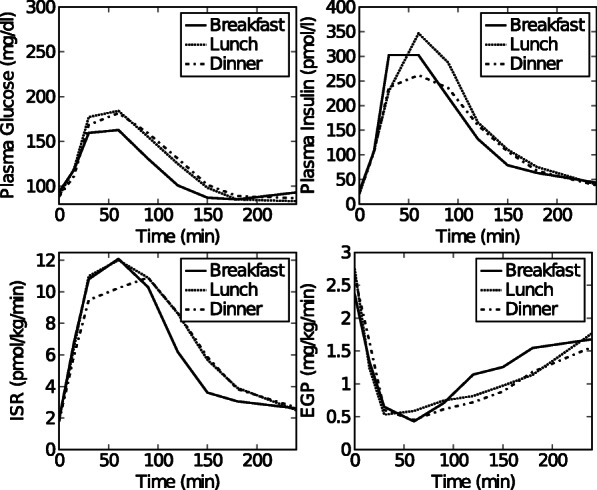
Dataset 1. Time courses of plasma glucose, plasma insulin, *ISR* and *EGP* after breakfast, lunch and dinner for a representative healthy subject. This data set was constructed from data in [28] as described in the text and in (Additional file 1: Figure S1)

Figure 2 shows the residual plots for each data set to indicate the model performance. Data set 1 showed the highest error (0.6 mg/kg/min) between the predicted EGP and experimental EGP for breakfast, lunch and dinner. The model showed both under and over estimation for this data set. Lower errors were observed for the data sets 2-5. The error range of the predicted EGP for data sets (2-5) was ±0.3 mg/kg/min. Remarkable is that the model systematically under estimated the data from data set 3. On the other hand, the model overestimated the EGP in data set 5.

### Reliability of parameter estimates

The parameter values for the data sets 1-5 were obtained without using the available experimental EGP data, however, this raised the question if these values were estimated reliably compared to the optimal parameter values obtained by fitting the Dalla Man [20] model to the experimental EGP data. To approach this question parameter identifiability analyses employing profile likelihood (PL) estimates were performed. Parameters were considered reliably estimated if they were located within the point-wise confidence interval.

**Fig. 3:**
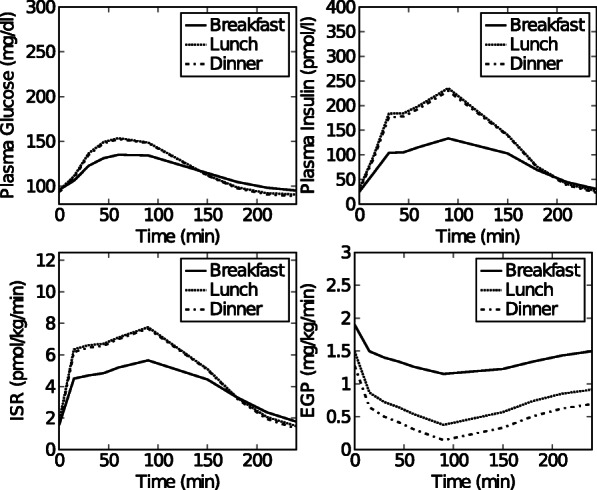
Dataset 2. Simulated time courses of plasma glucose, plasma insulin, *ISR* and *EGP* concentrations after breakfast, lunch and dinner for a normal, healthy subject obtained using the simulation model in [20]

The PL estimates for the parameter values (*k*_*p*2_, *k*_*p*3_, *k*_*p*4_ and *A*) are shown in Fig. 3 for data set 1-5. The resulting plots reveal practically non-identifiable and identifiable parameters. However, the observed systematic bias is negligible.

## Discussion

This study describes the successful development of a new procedure for the estimation of physiological parameters in a mathematical description of diurnal EGP dynamics in healthy, pre-diabetic and type 2 diabetic subjects. The procedure exploits the assumption that certain relations between the parameters do not vary between meals during the day. The mathematical description is based on a previously developed computational model describing glucose and insulin dynamics during a mixed meal [20]. A subsystem in this model, duly parameterized, can estimate the EGP using as input the plasma glucose, plasma insulin and portal insulin dynamics data. The various parameters of the subsystem in [20] are estimated from independently determined EGP dynamics, which requires glucose isotope labeling experiments. The proposed procedure obviates the need for such isotope experiments because it can estimate the model parameters directly from data on plasma glucose, insulin and *ISR* dynamics assembled during 3 consecutive meals.

Several articles reported that the insulin sensitivity is different in the morning (breakfast) compared with the rest of the day or evening [24, 27–29]. To take this diurnal effect into account, we multiplied insulin sensitivity during breakfast vs. the other meals by a factor *A*. Obtained values of *A* were between 0.8 and 0.9 for the different data sets. Interestingly, directly fitting the experimental EGP data against the model also yielded an optimal *A* value smaller than 1 for data set 1.

In the procedure proposed in the present paper, basal EGP value (Eq. 5) was not predicted, but was given to the model as a constant value extracted from the experimental EGP data (i.e., value at t = 0). As a future perspective, a separate prediction procedure could be developed for basal EGP.

The new procedure yielded estimates for the parameters (*k*_*p*2_, *k*_*p*3_, *k*_*p*4_ and *A*) on the basis of which the EGP dynamics were calculated using the model equation [20]. In general the estimated parameter values for the 5 individual data sets showed some deviations compared to the true values. The PL was therefore used to determine the reliability of the obtained parameter values for the different data sets. The analysis demonstrated that a systematic bias is negligible and that most parameters values were reliably estimated because they were located within the defined confidence interval.

One interesting point is that parameter differences between the healthy data sets (data sets 1 and 2) and the diabetic data set (data set 5) were not clearly observed. In fact, while Dalla Man [20] report different values for diabetic vs healthy datasets [20], no standard deviations are reported so one cannot judge the statistical significance of these differences. In the present study, the estimated parameters for data sets 1 and 2 and the diabetic data set (data set 5) may well not differ because they are depending on the particular input data from single individuals. From the PL analysis it can be appreciated that this is indeed the case, as judged from the fairly large confidence intervals around the estimated parameter values. It may still well be possible that when comparing results for groups of individuals, the mean estimated parameter values for the healthy and diabetes groups will show significant differences but this requires a separate, more exhaustive study.

The inaccuracy of the predicted EGP in data set 1, which was extracted from experimental data on healthy subjects, was higher compared to the other data sets (data sets 3-5), as judged from the residuals and R2 values. The model showed to systematically overestimate the EGP in data set 5. This indicates that the predicted EGP reflects a healthier condition than the experimental EGP. However, the maximal error range is rather small with 0.3 mg/kg/min. Thus, despite the deviations observed for the parameter estimates, the procedure showed a generally accurate prediction of the EGP dynamics for 3 consecutive meals.

In theory, our procedure has broader relevance for a wider spectrum of physiological models. Notably, the procedure might be transferable to models wherein a time-dependent variable (here: EGP) cannot be directly measured whereas a deterministic set of equations is available that relates this variable to other time-dependent variables (here: *G*_*p*_, *I*_*d*_, and *I*_*po*_) that are measurable.

In this study, five data sets were available for the development of the new and promising methodology to determine EGP dynamics. Only one data set contained experimental diurnal EGP dynamics. However, since the associated experimental errors were not given, it could not be assessed whether the predicted EGP was within the bandwidth set by the experimental inaccuracy. Therefore, it is difficult to precisely judge the quality of the EGP prediction in this procedure. In general, an experimental inaccuracy could be caused by recycling of labeled glucose via glycogenolysis and/or gluconeogenesis, and the equilibration of the tracer/tracee ratio [30]. A next step towards application of the new method in clinical studies will therefore the search for, and possible de novo acquisition, of multiple individual experimental data sets of different subtypes (healthy, pre-diabetic, T2D subjects), however this is subject of a separate investigation.

## Conclusions

In conclusion, a procedure was developed to retrieve physiologically based parameters for the description of diurnal EGP dynamics in subjects without the use of tracer techniques. The procedure makes use of input data consisting of diurnal dynamics of plasma glucose, insulin and *ISR*. The developed procedure accurately predicted (with an overall mean *R*^2^ of 0.9) diurnal EGP dynamics data for one experimental and four simulated data sets of healthy, pre-diabetic and type 2 diabetic subjects. The advantage is that diurnal EGP dynamics can be personally predicted. In the long run, more experimental data from healthy, pre-diabetic, and type 2 diabetic subjects could contribute to further and extensive validation of the procedure required for clinical applications.

## Methods

### Computer software

Published data in graphical format was digitized for use in the present paper using the accurate data extraction program GraphClick [21, 22]. All model equations were implemented and analyzed in MATLAB (MATLAB, Version R2012, The MathWorks, Inc., Natick, Massachusetts, United States) (Additional file 2). Ordinary differential equations were simulated using the variable step solver *ode15s*. The insulin secretion rate (*ISR*) data were calculated from deconvolution of the C-peptide time course data using the software program ISEC [23].

### Published equations for Endogenous Glucose Production (EGP)

**Fig. 4:**
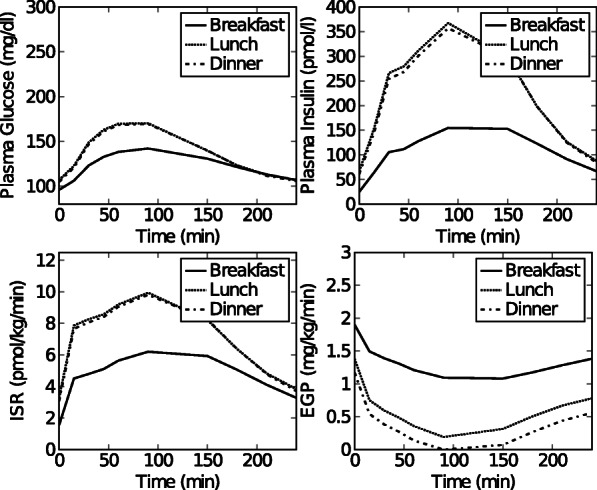
Dataset 3. Simulated time courses of plasma glucose, plasma insulin, *ISR* and *EGP* concentrations after breakfast, lunch and dinner for a glucose intolerant subject obtained using the simulation model in [20]

The objective was to develop a method for predicting the parameters of Endogenous Glucose Production (*EGP*) dynamics in the Dalla Man [20] model from experimental data without needing to apply isotope tracer experiments. The EGP dynamics after a meal in Dalla Man [20] are expressed as a function of glucose and insulin concentrations as schematically shown in Fig. 4.

From this model, the liver subsystem equations were used to formulate a set of equations to calculate *EGP* dynamics without the need of tracer experiments. From these *EGP* equations, partial derivatives were formulated and applied to calculate the liver subsystem parameters for estimating diurnal *EGP* dynamics as explained below.

In the following, the Dalla Man [20] equations (Eqs. 1–5) for the liver subsystem are repeated for convenience. The *EGP* is described as a function of inputs *G*_*p*_ (mg/kg) (glucose mass in plasma), *I*_*d*_ (pmol/l) (a delayed insulin signal) and *I*_*po*_ (pmol/kg) (insulin in the portal vein) using Eq. 1:

1 $$ EGP(t) = k_{p1} - k_{p2} \cdot G_{p}(t) - k_{p3} \cdot I_{d}(t) - k_{p4} \cdot I_{po}(t),  $$

wherein parameter *k*_*p*1_ (mg/kg/min) is the extrapolated *EGP* at zero glucose and insulin, *k*_*p*2_ (min^-1^) is liver glucose effectiveness, *k*_*p*3_ (mg/kg/min per pmol/l) is a parameter controlling the amplitude of insulin action on the liver, and *k*_*p*4_ (mg/kg/min per pmol/kg) is a parameter determining the amplitude of portal insulin action on the liver.

The glucose mass in plasma *G*_*p*_ is described using Eq. 2:

2 $$ G_{p} = G(t) * V_{g},  $$

wherein *V*_*g*_ is the distribution volume of glucose set to 1.88 dl/kg (healthy) or 1.49 dl/kg (type 2 diabetic) and *G*(*t*) is the experimental plasma glucose concentration (mg/dl) in time.

The delayed insulin signal, *I*_*d*_, is described using a chain of two-compartments:

3 $$ \begin{aligned} \dot{I_{1}}(t) &= -k_{i} \cdot (I_{1}(t) - I(t))&\qquad I_{1}(0) = I_{b} \\ \dot{I_{d}}(t) &= -k_{i} \cdot (I_{d}(t) - I_{1}(t)) &\qquad I_{d}(0) = I_{b}, \end{aligned}  $$

wherein *I* is the plasma insulin concentration (pmol/l) and the rate parameter *k*_*i*_ (=0.0079 min^-1^ (healthy) or 0.0066 min^-1^ (type 2 diabetic) from [20]) determines the delay between the insulin signal and the insulin action.

The portal insulin *I*_*po*_(pmol/kg) dynamics is described by

4 $$ I_{po} = \frac{ISR(t)}{\gamma},  $$

wherein the pancreatic insulin secretion rate (*ISR*(*t*) (pmol/kg/min)) can be calculated from experimental C-peptide data (details in “Experimental data set: healthy subject” section), and parameter *γ* (=0.5 min^-1^) is the fixed transfer rate constant between portal vein and liver.

Parameter *k*_*p*1_ is calculated as:

5 $$ k_{p1} = EGP_{\text{basal}} + k_{p2} \cdot G_{p, \text{basal}} + k_{p3} \cdot I_{d, \text{basal}} + k_{p4} \cdot I_{po, \text{basal}},  $$

where the basal data values for *EGP*, *G*_*p*_, *I*_*d*_, and *I*_*po*_ were extracted from experimental/simulated data sets (details in “Data” section) and *k*_*p*2_, *k*_*p*3_, *k*_*p*4_, and *A* were estimated as described in the following.

### Development of an objective function based on partial derivatives to estimate model parameters

Analyzing the different partial derivatives of Eq. 1 shows interesting relationships. For example, the partial differentiation ($\frac {\partial {EGP}}{\partial {k_{p2}}}$) is to first order equal to *G*_*p*_, ($\frac {\partial {EGP}}{\partial {k_{p3}}}$) to *I*_*d*_, and ($\frac {\partial {EGP}}{\partial {k_{p4}}}$) to *I*_*po*_. This suggests that EGP dynamics might be directly derived from experimental data for *G*_*p*_, *I*_*d*_ and *I*_*po*_ dynamics. We investigated further based on this interesting observation, by recombining Eq. 1 to find an alternative way to obtain the liver subsystem model parameters i.e. by using experimental data for *G*_*p*_, *I*_*d*_, *I*_*po*_ available from diurnal data (3 meals) without the need for isotope tracer experiments.

Taking the partial derivative of Eq. 1, with respect to *k*_*p*2_, *k*_*p*3_ and *k*_*p*4_, results in Eqs. 6, 7, and 8, respectively:

6 $$ \frac{\partial{EGP^{i}(t)}}{\partial{k_{p2}}} = -G_{p}^{i}(t) + \frac{\partial{k_{p1}^{i}}}{\partial{k_{p2}}}  $$

7 $$ \frac{\partial{EGP^{i}(t)}}{\partial{k_{p3}}} = -I_{d}^{i}(t) + \frac{\partial{k_{p1}^{i}}}{\partial{k_{p3}}}  $$

8 $$ \frac{\partial{EGP^{i}(t)}}{\partial{k_{p4}}} = -I_{po}^{i}(t) + \frac{\partial{k_{p1}^{i}}}{\partial{k_{p4}}},  $$

where *i* denotes breakfast (b), lunch (l), or dinner (d).

During the day, biochemical and physiological mechanisms are changing [24]. Especially, a strong variation in glucose tolerance during the day has been observed in different studies [25, 26]. It has also been demonstrated that the insulin responses in healthy subjects are different in the morning (breakfast) compared to the evening (dinner) [27]. Therefore, we decided to introduce a change in the insulin sensitivity during breakfast meal dynamics. To make this assumption more likely to be true, we explicitly took into account the only known intraday variation in EGP regulation, i.e. by introduction of parameter A. Parameter *k*_*p*4_ is related to the portal insulin concentration, *I*_*po*_, therefore *k*_*p*4_ was multiplied by an additional parameter *A* only during breakfast dynamics:

9 $$ \frac{\partial{EGP^{b}(t)}}{\partial{k_{p4}^{*}}} = -I_{po}^{i}(t) + \frac{\partial{k_{p1}^{b}}}{\partial{k_{p4}^{*}}},  $$

where $k_{p4}^{*} = A \cdot k_{p4}$ only during breakfast meal dynamics (*EGP*^*b*^).

Multiplying Eq. 6 by *k*_*p*2_, Eq. 7 by *k*_*p*3_ and Eq. 8 by *k*_*p*4_ results in sensitivities, i.e. the change in *EGP* for normalized changes in the liver parameters, in Eqs. 10, 11, and 12:

10 $$ k_{p2} \cdot \frac{\partial{EGP^{i}(t)}}{\partial{k_{p2}}} = -k_{p2} \cdot {G_{p}}^{i}(t) + k_{p2} \cdot \frac{\partial{k_{p1}^{i}}}{\partial{k_{p2}}}  $$

11 $$ k_{p3} \cdot \frac{\partial{EGP^{i}(t)}}{\partial{k_{p3}}} = -k_{p3} \cdot {I_{d}}^{i}(t) + k_{p3} \cdot \frac{\partial{k_{p1}^{i}}}{\partial{k_{p3}}}  $$

12 $$ k_{p4} \cdot \frac{\partial{EGP^{i}(t)}}{\partial{k_{p4}}}= -k_{p4} \cdot {I_{po}}^{i}(t) + k_{p4} \cdot \frac{\partial{k_{p1}^{i}}}{\partial{k_{p4}}},  $$

Subtracting Eq. 11 from Eqs. 10, 12 from Eqs. 10, and 12 from Eq. 11, results in Eqs. 13, 14 and 15, respectively:

13 $$ {\begin{aligned} k_{p2} \cdot \frac{\partial{EGP^{i}(t)}}{\partial{k_{p2}}} &- k_{p3} \cdot \frac{\partial{EGP^{i}(t)}}{\partial{k_{p3}}} \\&= -[k_{p2} \cdot G_{p}^{i}(t) - k_{p3} \cdot I_{d}^{i}(t)] + C_{23}^{i}, \end{aligned}}  $$

wherein $C_{23}^{i} = k_{p2} \cdot \frac {\partial {k_{p1}}}{\partial {k_{p2}}}^{i} - k_{p3} \cdot \frac {\partial {k_{p1}}}{\partial {k_{p3}}}^{i}$

14 $$ {\begin{aligned} k_{p2} \cdot \frac{\partial{EGP^{i}(t)}}{\partial{k_{p2}}} &- k_{p4} \cdot \frac{\partial{EGP^{i}(t)}}{\partial{k_{p4}}} \\&= -[k_{p2} \cdot G_{p}^{i}(t) - k_{p4} \cdot I_{po}^{i}(t)] + C_{24}^{i}, \end{aligned}}  $$

wherein $C_{24}^{i} = k_{p2} \cdot \frac {\partial {k_{p1}}}{\partial {k_{p2}}}^{i} - k_{p4} \cdot \frac {\partial {k_{p1}}}{\partial {k_{p4}}}^{i}$

15 $$ {\begin{aligned} k_{p3} \cdot \frac{\partial{EGP^{i}(t)}}{\partial{k_{p3}}} &- k_{p4} \cdot \frac{\partial{EGP^{i}(t)}}{\partial{k_{p4}}} \\&= -[k_{p3} \cdot I_{d}^{i}(t) - k_{p4} \cdot I_{po}^{i}(t)] + C_{34}^{i} \end{aligned}}  $$

wherein $C_{34}^{i} = k_{p3} \cdot \frac {\partial {k_{p1}}}{\partial {k_{p3}}}^{i} - k_{p4} \cdot \frac {\partial {k_{p1}}}{\partial {k_{p4}}}^{i}$

The $C^{i}_{23}$, $C^{i}_{24}$, and $C^{i}_{34}$ values represent meal-specific weighted differences of sensitivities of *k*_*p*1_ (extrapolated EGP at zero glucose and insulin) vs. multiple parameter pairs (*k*_*p*2_ and *k*_*p*3_, *k*_*p*2_ and *k*_*p*4_, and *k*_*p*3_ and *k*_*p*4_, respectively).

To estimate single values for *k*_*p*2_, *k*_*p*3_, *k*_*p*4_, and *A* for a single subject it was assumed that the mathematical constructs called sensitivities ($C^{i}_{23}$, $C^{i}_{24}$, and $C^{i}_{34}$) do not vary between meals. These mathematical constructs themselves do not translate to simple biological concepts, however they relate parameters that are linked to regulation of EGP. In essence, the assumption is therefore that EGP regulation does not change between 3 successive meals, resulting in:

16 $$ C_{23}^{b} = C_{23}^{l} = C_{23}^{d}  $$

17 $$ C_{24}^{b} = C_{24}^{l} = C_{24}^{d}  $$

18 $$ C_{34}^{b} = C_{34}^{l} = C_{34}^{d}  $$

The model EGP dynamics objective function to obtain the parameter values of *k*_*p*2_, *k*_*p*3_, *k*_*p*4_, and *A* is therefore formulated by combining Eqs. 16, 17, 18:

19 $$ {\begin{aligned} \hat{\theta} = \text{arg min} \sqrt{\sum\limits_{i, j, i \neq j} \left(\left(C_{23}^{i} - C_{23}^{j}\right) + \left(C_{24}^{i} - C_{24}^{j}\right) + \left(C_{34}^{i} - C_{34}^{j}\right)\right)^{2}}, \end{aligned}}  $$

wherein *i* and *j* are any two combinations of breakfast (b), lunch (l) and dinner (d).

This methodology thus focuses on obtaining estimates only for the parameters which are directly involved in the general EGP dynamics equation (Eq. 1). The parameters *k*_*i*_, *V*_*g*_, and *γ* relating to glucose kinetics and insulin secretion processes are not directly in the EGP equation and therefore not fitted. However, although these parameters do not appear directly in the equation, they still influence the result of the EGP equation since they multiply/divide the values of measured quantities. An inaccurate assumption of their value could lead to a wrong estimation of the directly involved parameters *k*_*p*2_, *k*_*p*3_ and *k*_*p*4_. The model parameter values *k*_*i*_, *V*_*g*_, and *γ* were extracted from Dalla Man (Table 1) [20]. Here, they obtained these values for normal and type 2 diabetic subjects. So, if the fasting glucose value is below 99 mg/dl (5.5 mmol/l) then the Dalla Man parameter values (*k*_*i*_, *V*_*g*_, and *γ*) for normal subjects should be used. Parameter values of type 2 diabetic should be used if the fasting plasma glucose value is higher than 126 mg/dl (7.0 mmol/l). For fasting glucose values between 99 mg/dl (5.5 mmol/l) and 126 mg/dl (7 mmol/l) estimates for *k*_*i*_, *V*_*g*_, and *γ* can be obtained through linear interpolation of Dalla Man [20] normal, and type 2 diabetic subject parameter values.

Briefly, the derivation evolves around a liver-related parameter *k*_*p*1_ which itself explains a rather virtual concept, namely the extrapolated EGP at zero glucose and insulin. This parameter is used to describe the specific behavior of EGP in the virtual condition that no glucose and insulin were present. In the model EGP dynamics objective function, the sensitivity of this parameter to other parameters (i.e. proportional changes of *k*_*p*1_ in response to changes in parameters *k*_*p*2_, *k*_*p*3_ and *k*_*p*4_) are used. These terms originate in Eqs. 6–8 and appear weighted in Eqs. 10–12. Then, coefficients consisting of differences of pairs of these weighted terms are formed in Eqs. 13–15. The central assumption is that these coefficients do not differ between 3 subsequent meals as expressed in Eqs. 16–18. Hence, the parameter values for a single person are obtained and are equal for breakfast, lunch, and dinner. As stated earlier, the essence of the underlying biological hypothesis is that the parameters for regulation of hepatic EGP production do not vary during 3 consecutive meals within a single day, but the derivation follows a mathematical rather than a biological reasoning.

### Data

The procedure was applied to an experimental data set and simulated data sets (5 data sets in total) representing different health conditions of subjects as follows:

#### Experimental data set: healthy subject

**Data set 1: Healthy subject** Experimental mean 3-meal data curves of 20 healthy subjects (i.e. mean plasma glucose, plasma insulin, C-peptide and *EGP*) were extracted from Fig 1A, 1B, and 2A in [28] using GraphClick [22]. The data were converted to the desired units (Glucose: mmol/l to mg/dl (×18.02); *EGP*: *μ*mol/kg/min to mg/kg/min (×0.18)). Each segment (breakfast, lunch and dinner), starts when the meal is given at time zero, and ends 240 minutes later. The *ISR* was calculated from C-peptide data (Fig 1B in [28]) using the software program ISEC [23].

**Fig. 5:**
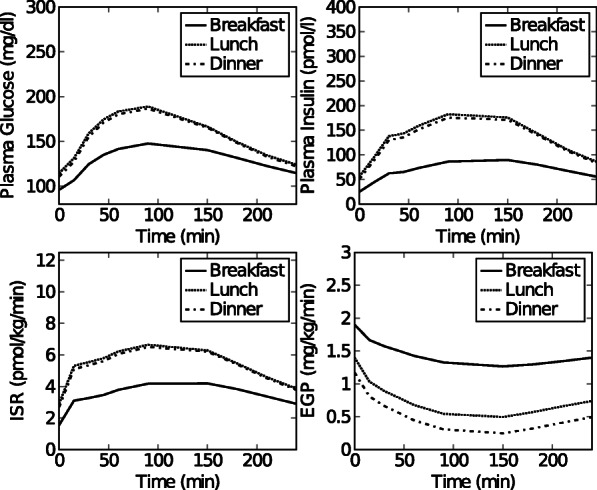
Dataset 4. Simulated time courses of plasma glucose, plasma insulin, *ISR* and *EGP* concentrations after breakfast, lunch and dinner for a subject with a decreased insulin action obtained using the simulation model in [20]

Preprocessing of the data set was necessary in order to obtain a single set of plasma glucose, plasma insulin and *ISR* data curves for a hypothetical representative individual subject in the study of [28] that were consistent with the mean EGP curves of the 20 subjects. We adopted this procedure because the individual data sets remained unavailable. The procedure is explained and the raw data can be seen in (Additional file 1). The result, i.e. the final input data for the model, is depicted in Fig. 5. Data was collected at time points 0, 15, 30, 45, 60, 90, 120, 150, 180, 210, 240 minutes. This data set is referred to as data set 1 throughout the paper.

#### Simulated data sets: healthy, pre-diabetic, and diabetic subjects

**Fig. 6:**
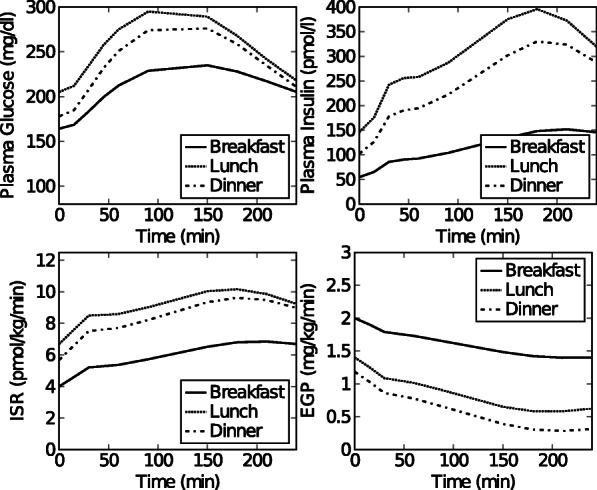
Dataset 5. Simulated time courses of plasma glucose, plasma insulin, *ISR* and *EGP* concentrations after breakfast, lunch and dinner for a type 2 diabetic subject obtained using the simulation model in [20]

**Data set 2: Healthy subject** Simulated 3-meal data curves (i.e. plasma glucose, plasma insulin, *ISR* and *EGP*) for a healthy subject were generated using the meal simulation model in [20]. To this end, the in silico model in [20] was implemented in MATLAB and the parametric portrait of a normal subject (Table 1 in [20]) was used for simulation.The predicted plasma glucose, plasma insulin, *ISR* and *EGP* were based on 3 consecutive meals i.e. a breakfast (8 a.m.) meal of 45 gram, a lunch (12 p.m.) meal of 70 gram, and a dinner (8 p.m.) meal of 70 gram. Each meal segment starts when the meal is given (t=0 for that segment) and ends 240 min later. Data was collected at time points 0, 15, 30, 45, 60, 90, 120, 150, 180, 210, 240 min. The data is referred to as data set 2 throughout the paper, and can be seen in Fig. 6.

**Fig. 7:**
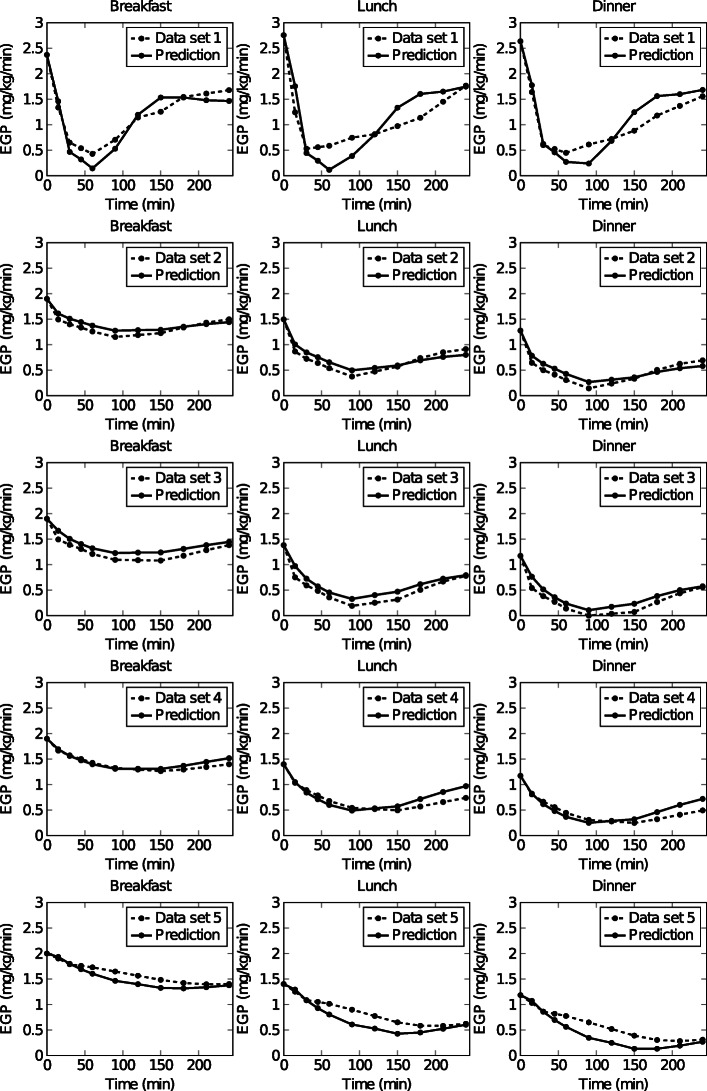
Predictions (solid line) versus measurements (dashed line) of EGP during breakfast, lunch and dinner for (top to bottom) data set 1, data set 2, data set 3, data set 4, and data set 5

**Fig. 8:**
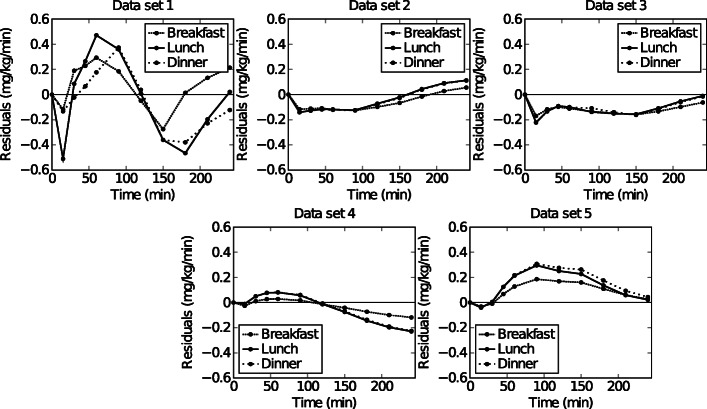
Residual analysis plot of EGP during breakfast, lunch and dinner for data set 1, data set 2, data set 3, data set 4, and data set 5 calculated by subtracting the predicted EGP from the experimental EGP

**Data sets 3 and 4: Pre-diabetic subjects** The in silico model in [20] implemented in MATLAB was used to obtain simulation data for 2 pre-diabetic subjects having different subtypes i.e.(1) glucose intolerance, and (2) decreased insulin action. For the glucose intolerant subject, the parameter values for normal subjects ([20], Table 1) were used, except that *V*_*max*_ and *k*_*p*3_, were halved. For the subject with decreased insulin action, parameter values for normal subjects were used except that *K* and *β* were halved. The predicted plasma glucose, plasma insulin, *ISR* and *EGP* were based on 3 consecutive meals i.e. a breakfast (8 a.m.) meal of 45 gram, a lunch (12 p.m.) meal of 70 gram, and a dinner (8 p.m.) meal of 70 gram. Each meal segment starts when the meal is given (t=0 for that segment) and ends 240 min later. Data was collected at time points 0, 15, 30, 45, 60, 90, 120, 150, 180, 210, 240 min. The data are referred to as data set 3 (glucose intolerant subject) and data set 4 (subject with a decreased insulin action) throughout the paper, and can be seen in Figs. 7 and 8.

**Fig. 9:**
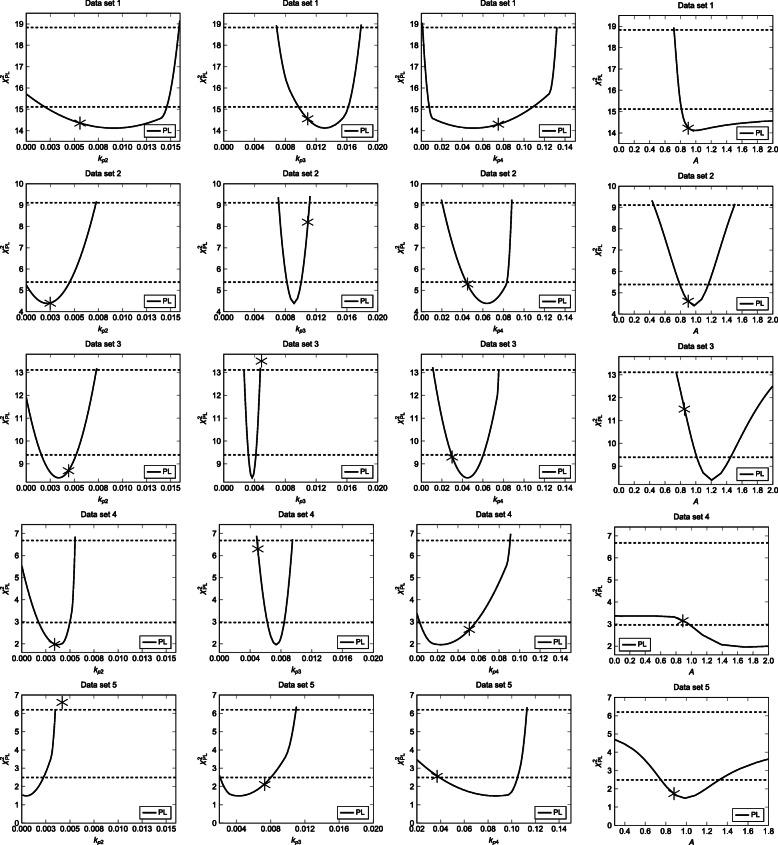
Profile likelihood estimates (Y-axis) (PL; continuous black line) vs. model parameters (X-axis) for the model with (top to bottom) data set 1, data set 2, data set 3, data set 4, and data set 5. The parameter estimates (cf. Table 1) obtained using the new approach are indicated by gray asteriks

**Data set 5: type 2 diabetic subject** The in silico model in [20] implemented in MATLAB was used to obtain simulation data for a type 2 diabetic subject. The parameter values for type 2 diabetic subjects ([20], Table 1) were used. The predicted plasma glucose, plasma insulin, *ISR* and *EGP* were based on 3 consecutive meals i.e. a breakfast (8 a.m.) meal of 45 gram, a lunch (12 p.m.) meal of 70 gram, and a dinner (8 p.m.) meal of 70 gram. Each meal segment starts when the meal is given (t=0 for that segment) and ends 240 min later. Data was collected at time points 0, 15, 30, 45, 60, 90, 120, 150, 180, 210, 240 min. The data are referred to as data set 5 throughout the paper, and can be seen in Fig. 9.

### Parameter optimization strategy

The EGP data fitting procedure was implemented in Matlab with the function *lsqnonlin*, minimizing the model EGP dynamics objective function (Eq. 19). Lower boundaries were set to 0.0009, 0.001, 0.01, 0.8 and upper boundaries to 0.02, 0.05, 0.09, 1.4 for *k*_*p*2_, *k*_*p*3_, *k*_*p*4_ and *A*, respectively. The function *multistart* was used to start the *lsqnonlin* function from multiple start points (150) in order to increase the probability of finding a global optimum. The reported parameters for each data set were those for which the model EGP dynamics objective function achieved its minimal value.

The obtained parameter values for each data set were also checked against the optimal values, which are determined from directly fitting the experimental EGP against the model. These optimal parameter values were calculated by minimizing the following cost function (V) with the above explained settings:

20 $$ V(p) = \sqrt{(EGP_{i,j}^{calc}(p,t) - EGP_{i,j}^{exp}(t))^{2}},  $$

wherein the cost function V is defined as the squared difference between the model output ($EGP^{calc}_{i,j}$) and experimental *EGP* ($EGP^{exp}_{i,j}$) for every time step, *i*, the model parameters *p*, and for breakfast, lunch and dinner, *j*. Here 5 data sets that originated from the experimental and simulated data (Figs. 5, 6, 7, 8 and 9) were treated as experimental data ($EGP^{exp}_{i,j}$).

### Parameter identifiability analysis

To investigate the reliability of the obtained parameter estimates (*k*_*p*2_, *k*_*p*3_, *k*_*p*4_ and *A*), parameter identifiability analysis using PL [31] was performed. PL allows to derive the identifiability of parameters in non-linear models, to design optimal experiments that improve parameter identification and to calculate likelihood-based confidence intervals.

First, the weighted sum of squared residuals was set as the PL objective function measuring the agreement of experimental data with model-predicted data:

21 $$ \chi^{2}(\theta) = \sum_{k=1}^{m} \sum_{l=1}^{d} \left(\frac{y^{exp}_{kl} - y^{cal}_{kl} (\theta)}{\sigma^{exp}_{kl}} \right)^{2}  $$

where $y^{exp}_{kl}$ denotes the experimental EGP data for each observable time point *k*, $\sigma ^{exp}_{kl}$ is the measurement error of the experimental data and was assumed to be normally distributed. Here 5 data sets that originated from the experimental and simulated data (Figs. 5, 6, 7, 8 and 9) were treated as experimental data $\left (y^{exp}_{kl}\right)$.

The $y^{cal}_{k}$ are calculated EGP values at time points when experimental data were measured, *m* is the number of time points and *d* is the number of meal segments.

In the PL procedure, the parameters were calculated numerically:

22 $$ \hat{\theta} = {\text{arg min}} \left[ \chi^{2}(\theta) \right],  $$

where the value $\chi ^{2}(\hat {\theta })$ corresponds to the maximum likelihood estimation for Gaussian measurement noise. The PL is determined by an iterative procedure where the first of the parameters is slightly shifted from its optimal value [31, 32] whereafter the other parameters are fitted again. Then the parameter is shifted again and the other parameters are fitted again. The shifting is done both towards smaller values, and towards higher values. This procedure for a given parameter will end in either direction when a threshold in the likelihood is met. After that, the procedure is repeated for the other parameters. The likelihood-based confidence interval for a parameter was calculated to define a confidence region [31, 33]:

23 $$ \{\theta \mid \chi^{2}(\theta) - \chi^{2}(\hat{\theta}) < \Delta_{\alpha} \} \quad \text{with} \quad \Delta_{\alpha} = \chi^{2}(\alpha, df),  $$

with df the number of degrees of freedom and *α* the confidence level. The df gives for df=1 the point-wise and for df=4 (number of fitted parameters) the simultaneous confidence interval, both to the confidence level *α* of 0.68, which corresponds to one standard deviation.

A parameter is identifiable if the confidence interval [ *σ*^−^, *σ*^+^] of its estimate $\hat {\theta }$ is finite. This indicates that this parameter can be calculated from experimental data sets. Practical non-identifiability occurs when a parameter shows a likelihood-based confidence region which is infinite extended in the direction of *θ*, even if the likelihood has a unique minimum for this parameter [31]. The confidence interval of a practically non-identifiable parameter is not automatically infinitely extended to both sides; a finite lower or upper bound of the confidence interval [ *σ*^−^, *σ*^+^] can still exist. Resolving a practically non-identifiable parameter is possible by increasing the amount and quality of experimental data and/or the choice of the time points [31]. Structural non-identifiability occurs if the confidence interval is infinite to both sides. Structural non-identifiability cannot be solved by a more accurate experimental data set, and indicates that there is missing information [31].

With this method, obtained parameter values were inspected on the deviations from $\chi ^{2}(\hat {\theta })$ and if the corresponding predicted model observable ($y^{cal}_{kl}$) was in agreement with experimental noise, assumed to be $y^{exp}_{kl}*0.1 + \text {max}(y^{exp}_{kl})*0.05$, so as to gain information on the quality of the model predictions.

## Supplementary information

**Preprocessing of data set 1 (experimental data set).** Ref. [28] gives mean data input curves of 20 subjects. To test the new procedure developed in this paper, a set of data curves for plasma glucose ( *G*_*p*_), plasma insulin ( *I*_*p*_) and insulin secretion rate (*ISR*) for a *single subject* needed to be generated that would produce the mean *EGP* data curves of the 20 subjects. To this end, the parameters (p), *k*_*p*2_, *k*_*p*3_ and *k*_*p*4_ plus additional parameters representing the mean input data (i.e. *G*_*p*_, *I*_*p*_ and *ISR*) at time points 20, 30, 60, and 90 minutes for 3 meals (see below), were optimized using the gradient-based least squares solver *lsqnonlin* in MATLAB (see Additional file 1: Figure S1). This was performed by minimizing the preprocessing experimental data objective function (Eq. 24):
$ Obj (p) = \sqrt {(EGP_{i,j}^{calc}(p,t) - EGP_{i,j}^{exp}(t))^{2}}, \qquad \text {(24)} $
wherein the function Obj is defined as the squared difference between the Dalla Man [20] model output ($EGP^{calc}_{i,j}$) and experimental *EGP* ($EGP^{exp}_{i,j}$) for every time step, *i*, and for breakfast, lunch and dinner, *j*. For experimental data ($EGP^{exp}_{i,j}$) in Eq. 24 is taken from Saad et al. [28].The additional parameters for single-subject *G*_*p*_, *I*_*p*_, and *ISR* at t=20, 30, 60, and 90 minutes were defined as the shifts from the mean data values and were constrained within ±10 mg/dl, ±25 pmol/l, and ±1 pmol/kg/min, respectively. Additional file 1: Table S1 displays the values for these shifts that resulted from the optimization procedure. The resulting single-subject data were used as data set 1 (Fig. 2).

**Additional file 2** mATLAB code. MATLAB code of the described methodology.

## Data Availability

All data and software generated or analysed during this study are included in this published article and its supplementary information files.

## Abbreviations

df: Degrees of freedom
EGP: Endogenous glucose production
ISR: Insulin secretion rate
PL: Profile likelihood
T2D: Type 2 diabetes
