# Supplementary figures and images for: Determination of physiological parameters for endogenous glucose production in individuals using diurnal data
Source: BMC Biomed Eng. 2019 Nov 15;1:29. doi: 10.1186/s42490-019-0030-z (PMC7422590; doi:10.1186/s42490-019-0030-z)

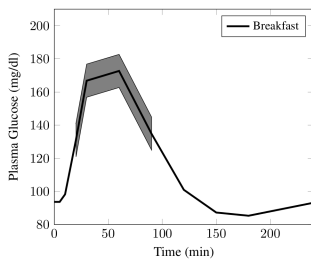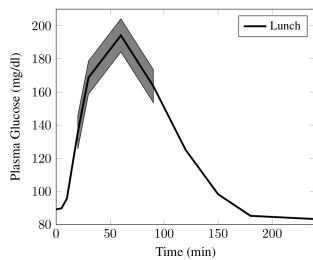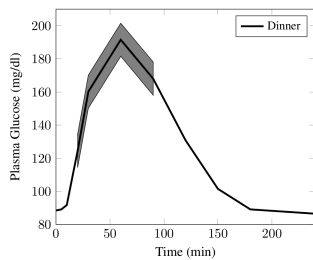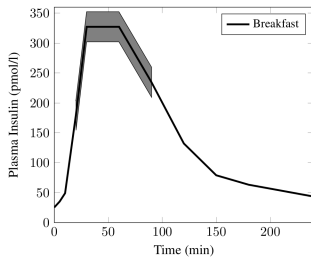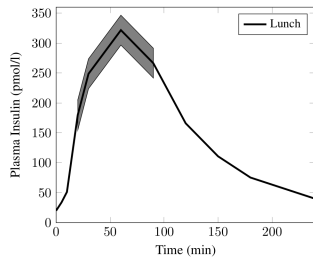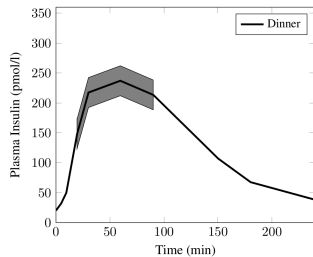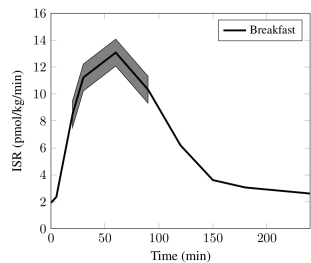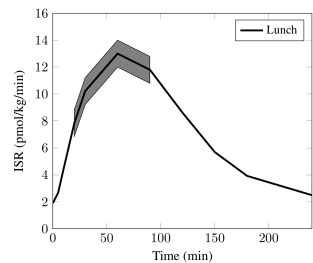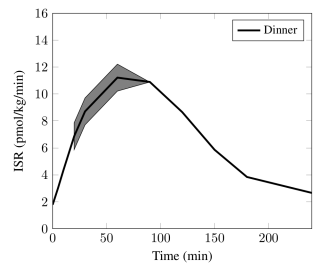

Supplement: Supplementary file 1 — Preprocessing of data set 1 (experimental data set). Ref. [28] gives mean data input curves of 20 subjects. To test the new procedure developed in this paper, a set of data curves for plasma glucose ( Gp), plasma insulin ( Ip) and insulin secretion rate (ISR) for a single subject needed to be generated that would produce the mean EGP data curves of the 20 subjects. To this end, the parameters (p), kp2, kp3 and kp4 plus additional parameters representing the mean input data (i.e. Gp, Ip and ISR) at time points 20, 30, 60, and 90 minutes for 3 meals (see below), were optimized using the gradient-based least squares solver lsqnonlin in MATLAB (see Additional file 1: Figure S1). This was performed by minimizing the preprocessing experimental data objective function (Eq. 24): $Obj (p) = \sqrt {(EGP_{i,j}^{calc}(p,t) - EGP_{i,j}^{exp}(t))^{2}}, \qquad \text {(24)}$Obj(p)=(EGPi,jcalc(p,t)−EGPi,jexp(t))2,(24) wherein the function Obj is defined as the squared difference between the Dalla Man [20] model output ($EGP^{calc}_{i,j}$EGPi,jcalc) and experimental EGP ($EGP^{exp}_{i,j}$EGPi,jexp) for every time step, i, and for breakfast, lunch and dinner, j. For experimental data ( [file 42490_2019_30_MOESM1_ESM.pdf]
