# Supplementary material for: Determination of physiological parameters for endogenous glucose production in individuals using diurnal data
Source: BMC Biomed Eng. 2019 Nov 15;1:29. doi: 10.1186/s42490-019-0030-z (PMC7422590; doi:10.1186/s42490-019-0030-z)
Supplement: Supplementary file 2 — Additional file 2 mATLAB code. MATLAB code of the described methodology. [file 42490_2019_30_MOESM2_ESM.pdf]

```
function optSsLiver_optim_START()

%% Structuring of the selected dataset

%dataset1
% load('Saad12meals_opt.mat');
% [t, t1, t2, G1, I, S, EGPM, Gb, Ib, Sb] = inputdata(Saad12breakfastCopy, Saad12lunchCopy, Saad12dinnerCopy);

%dataset2
   load('24h_normalsubjects', 'DM_B_normal', 'DM_L_normal', 'DM_D_normal');
   [t, t1, t2, G1, I, S, EGPM, Gb, Ib, Sb] = inputdata(DM_B_normal, DM_L_normal, DM_D_normal);

%dataset3
%   load('24h_GIsubjects', 'DM_B_GIS_tshort', 'DM_L_GIS_tshort', 'DM_D_GIS_tshort')
%    [t, t1, t2, G1, I, S, EGPM, Gb, Ib, Sb] = inputdata(DM_B_GIS_tshort, DM_L_GIS_tshort, DM_D_GIS_tshort);

%dataset4
%   load('24h_halveKandB', 'DM_B_halveKandB_tshort', 'DM_L_halveKandB_tshort', 'DM_D_halveKandB_tshort');
%   [t, t1, t2, G1, I, S, EGPM, Gb, Ib, Sb] = inputdata(DM_B_halveKandB_tshort, DM_L_halveKandB_tshort, ...
%       DM_D_halveKandB_tshort);

%dataset5
%   load('24h_diabeticsubjects', 'DM_B_diabetic_tshort', 'DM_L_diabetic_tshort', 'DM_D_diabetic_tshort')
%   [t, t1, t2, G1, I, S, EGPM, Gb, Ib, Sb] = inputdata(DM_B_diabetic_tshort, DM_L_diabetic_tshort, ...
%       DM_D_diabetic_tshort);

%% Set initial parameter values

p1(1,1) = 0.0056;    % kp2
p1(2,1) = 0.0109;    % kp3
p1(3,1) = 0.075;     % kp4
p1(4,1) = 0.9;       % alpha

lb1 = [0.0009; 0.001;0.01; 0.8];
ub1 = [0.02; 0.05; 0.09; 1.4];

options = optimoptions(@lsqnonlin,'Algorithm','trust-region-reflective', 'MaxIterations', 30,...
    'Display', 'Iter','TolX',1e-10,'TolFun',1e-10)

%% Optimize problem

problem = createOptimProblem('lsqnonlin','objective', @(p1)optSsLiver_optim(p1, t, t1, t2, G1, I, S, EGPM, Gb, Ib, Sb),...
'x0', p1(), 'lb', lb1, 'ub', ub1, 'options', options);

ms = MultiStart;

[param,~] = run(ms,problem,150);

end

function   Matrix = optSsLiver_optim(p1, t, t1, t2, G1, I, S, EGPM, Gb, Ib, Sb)

%% Delta value
delta = 5e-5;

%% Kp2 and kp3
% set value parameters kp2, kp3 and kp4
p(1,:)    =   [p1(1,1), p1(1,1)+delta, p1(1,1)];     % kp2
p(2,:)    =   [p1(2,1), p1(2,1), p1(2,1)+delta];     % kp3
p(3,:)    =   [p1(3,1), p1(3,1), p1(3,1)];           % kp4
p(4,:)    =   [p1(4,1), p1(4,1), p1(4,1)];           % alpha

% Calculate EGP procedure
% output consists of EGP, Gp, Il, Id, Ipo, kp1
output1 = optprocLiver(p(:,1),t, t1, t2, G1, I, S, EGPM, Gb, Ib, Sb, 1);
output2 = optprocLiver(p(:,2),t, t1, t2, G1, I, S, EGPM, Gb, Ib, Sb, 1);
output3 = optprocLiver(p(:,3),t, t1, t2, G1, I, S, EGPM, Gb, Ib, Sb, 1);

% calculate sensitivity
[diffC1] = calcSens(output1, output2, output3, p);

%% Kp2 and kp4
clear output1 output2 output3 p

% set value parameters kp2, kp3 and kp4
p(1,:)    =   [p1(1,1), p1(1,1)+delta, p1(1,1)];     % kp2
p(2,:)    =   [p1(2,1), p1(2,1), p1(2,1)];           % kp3
p(3,:)    =   [p1(3,1), p1(3,1), p1(3,1)+delta];     % kp4
p(4,:)    =   [p1(4,1), p1(4,1), p1(4,1)];           % alpha

% Optimization procedure
% output consists of EGP, Gp, Il, Id, Ipo, kp1
output1 = optprocLiver(p(:,1),t, t1, t2, G1, I, S, EGPM, Gb, Ib, Sb, 1);
output2 = optprocLiver(p(:,2),t, t1, t2, G1, I, S, EGPM, Gb, Ib, Sb, 1);
output3 = optprocLiver(p(:,3),t, t1, t2, G1, I, S, EGPM, Gb, Ib, Sb, 1);

% calculate sensitivity
[diffC2] = calcSenskp2kp4(output1, output2, output3, p);

%% Kp3 and kp4
```

```
clear output1 output2 output3 p

% set value parameters kp2, kp3 and kp4
p(1,:)    =   [p1(1,1), p1(1,1), p1(1,1)];          % kp2
p(2,:)    =   [p1(2,1), p1(2,1)+delta, p1(2,1)];    % kp3
p(3,:)    =   [p1(3,1), p1(3,1), p1(3,1)+delta];    % kp4
p(4,:)    =   [p1(4,1), p1(4,1), p1(4,1)];          % alpha

% optimization procedure
% output consists of EGP, Gp, Il, Id, Ipo, kp1
output1 = optprocLiver(p(:,1),t, t1, t2, G1, I, S, EGPM, Gb, Ib, Sb, 1);
output2 = optprocLiver(p(:,2),t, t1, t2, G1, I, S, EGPM, Gb, Ib, Sb, 1);
output3 = optprocLiver(p(:,3),t, t1, t2, G1, I, S, EGPM, Gb, Ib, Sb, 1);

% calculate sensitivity
[diffC3] = calcSenskp3kp4(output1, output2, output3, p);


%% Objective function
Matrix = [];

Obj = (diffC1(1,:)+diffC2(1,:)+diffC3(1,:)).^0.5;

Matrix = [Obj;Obj;Obj;Obj];

end

function [diffC] = calcSens(output1, output2, output3, p)

%% Sensitivity

dkp2 = p(1,1)-p(1,2);
dkp3 = p(2,1)-p(2,3);

%% Sensitivity C equation

dkp1kp2 = output1.kp1.val-output2.kp1.val;
dkp1kp3 = output1.kp1.val-output3.kp1.val;

C = p(1,1).* (dkp1kp2./dkp2) - p(2,1).* (dkp1kp3./dkp3);

diffC = [((C(1,1)-C(1,2)).^2), ((C(1,1)-C(1,3)).^2),((C(1,2)-C(1,3)).^2)];

end


function [diffC] = calcSenskp2kp4(output1, output2, output3, p)

%% Sensitivity

dkp2 = p(1,1)-p(1,2);
dkp4 = [(p(3,1).*(p(4,1)))-(p(3,3).*(p(4,1))), p(3,1)-p(3,3),p(3,1)-p(3,3)];

for i = 1:size(output1.kp1.val,1); dkp4(i,:) = dkp4(1,1:3); end

%% Sensitivity C equation
dkp1kp2 = output1.kp1.val-output2.kp1.val;
dkp1kp4 = output1.kp1.val-output3.kp1.val;

C1 = p(1,1).* (dkp1kp2./dkp2) - p(3,1).* (dkp1kp4./dkp4);
C = [p(1,1).* (dkp1kp2(:,1)./dkp2) - p(3,1).* (dkp1kp4(:,1)./dkp4(:,1)).*(1/p(4,1)), C1(:,2), C1(:,3)];

diffC = [((C(1,1)-C(1,2)).^2), ((C(1,1)-C(1,3)).^2),((C(1,2)-C(1,3)).^2) ];

end


function [diffC] = calcSenskp3kp4(output1, output2, output3, p)

%% Sensitivity

dkp3 = p(2,1)-p(2,2);
dkp4 = [(p(3,1).*(p(4,1)))-(p(3,3).*(p(4,1))), p(3,1)-p(3,3),p(3,1)-p(3,3)];

for i = 1:size(output1.kp1.val,1); dkp4(i,:) = dkp4(1,1:3);end

%% Sensitivity C equation

dkp1kp3 = output1.kp1.val-output2.kp1.val;
dkp1kp4 = output1.kp1.val-output3.kp1.val;

C1 = p(2,1).* (dkp1kp3./dkp3) - p(3,1).* (dkp1kp4./dkp4);
C = [p(2,1).* (dkp1kp3(:,1)./dkp3) - p(3,1).* (dkp1kp4(:,1)./dkp4(:,1)).*(1/p(4,1)), C1(:,2), C1(:,3)];


diffC = [((C(1,1)-C(1,2)).^2), ((C(1,1)-C(1,3)).^2),((C(1,2)-C(1,3)).^2)];

end
```

```
function [output] = optprocLiver(p,t, t1, t2, G1, I, S, EGPM, Gb, Ib, Sb, plt)

names(1).val    = 'kp2';
names(2).val    = 'kp3';
names(3).val    = 'kp4';
names(4).val    = 'alpha';

%% Basal values
pB(1,:)           = [Gb(1,1), Gb(1,2), Gb(1,3)];
pB(2,:)           = [Ib(1,1),Ib(1,2), Ib(1,3)];
pB(3,:)           = [Sb(1,1), Sb(1,2), Sb(1,3)];
pB(4,:)           = [EGPM(1,2), EGPM(1,3), EGPM(1,4)];

namesB(1).val   = 'Gb';
namesB(2).val   = 'Ib';
namesB(3).val   = 'Sb';
namesB(4).val   = 'EGP_b';


for k=1:length(names)
    par.(names(k).val).val = p(k,:);
end
for k=1:length(namesB)
    par.(namesB(k).val).val = pB(k,:);
end

%% Parameter constants
% Healthy
par.v_g.val     = 1.88;
par.ki.val      = 0.0079;
par.gam.val     = 0.5;

if plt
output = objFunLiver(p, par, names, t, G1, t1, I, t2, S, [], 0, EGPM);
else

end

end


function [fi] = objFunLiver(p, par, names, t0, G1, t1, I, t2, S, EGPsd, plt,EGPM)

% Fill "par" with current values
for k=1:length(p)
    par.(names(k).val).val = p(k);
end

% Calculate order, variables and initial conditions
o           = state_order_DM();
G           = G1;

%% Calculate interpolation functions
p_g.t       = t0(:,1);
p_g.g       = G;

p_g_dot.t   = (p_g.t(2:end)- p_g.t(1:end-1))/2 + p_g.t(1:end-1);

for i = 1:3     % 3 datasets
    p_g_dot.dot(:,i) = (p_g.g(2:end,i)- p_g.g(1:end-1,i)) ./ (p_g.t(2:end)- p_g.t(1:end-1));
end

p_i.t       = t1(:,1);
p_i.i       = I;


t = p_g.t;

% DETERMINE IS
i_s.t       = t2(:,1);
i_s.s       = S; % At same time point t

% i. Liver unit process model
var         = init_DM_i(par,o.i);
[ti,xi]     = tl_ode15s(@ode_DM_i, t, var.x0 ,[], par, var, o.i, p_g, p_i, i_s);
[si]               = states_DM_i(ti, xi, par, var, o.i);
[fi]        = flux_DM_i_LAST(ti, si, par, var, p_g, p_i, i_s);

td = [t,t,t];
Gu = G.*par.v_g.val;
Ipou = S./par.gam.val;


end

function [var] = init_DM_i(par, o)

par.kp4.val = [par.kp4.val.*(par.alpha.val), par.kp4.val, par.kp4.val];

% Variables
```

```
var.Ipob.val    =   par.Sb.val./par.gam.val;
var.Gpb.val     =   par.Gb.val.*par.v_g.val;
var.kp1.val     =   par.EGP_b.val + par.kp2.val.*var.Gpb.val + par.kp3.val.*par.Ib.val + par.kp4.val.*var.Ipob.val;

% Initial conditions
s0.I1           =   par.Ib.val;
s0.Id           =   par.Ib.val;

x0 = [s0.I1,s0.Id];

var.x0 = x0;
end


function [f] = flux_DM_i_LAST(t, s, par, var, p_g, p_i, i_s)

% Plasma G, I and insulin S -> EGP

% States:
% Id, I1,
% Parameters:
% v_g, k_i, kp2, kp3, kp4
% Variables:
% kp1
% Constants:
% gam

% Prerequisites for kp2
G           = interp1(p_g.t, p_g.g, t);
Gp          = G .* par.v_g.val;
% Prerequisites for kp3
I           = interp1(p_i.t, p_i.i, t);
f.I1_in.val = par.ki.val.*I;
f.I1_out.val= par.ki.val.*s.I1.val;
f.Id_out.val= par.ki.val.*s.Id.val;

% Prerequisites for kp4
if t >= i_s.t(1)
    S           = interp1(i_s.t, i_s.s, t);
else
    S           = i_s.s(1);
end

Ipo         = S ./ par.gam.val;
f.Ipo.val   = Ipo;

% Calculate EGP
par.kp4.val = [par.kp4.val.*(par.alpha.val), par.kp4.val, par.kp4.val];
for i = 1:length(Gp); var.kp1.val(i,:) = var.kp1.val(1,1:3);par.kp4.val(i,:) = par.kp4.val(1,1:3);end
f.EGP.val   = var.kp1.val - par.kp2.val.*Gp - par.kp3.val.*s.Id.val - par.kp4.val.*Ipo;

f.Id.val = s.Id.val;
f.kp1.val = var.kp1.val;

end
f.Gp.val = Gp;
```
